# Methanogen Diversity in Indigenous and Introduced Ruminant Species on the Tibetan Plateau

**DOI:** 10.1155/2016/5916067

**Published:** 2016-04-28

**Authors:** Xiao Dan Huang, Gonzalo Martinez-Fernandez, Jagadish Padmanabha, Ruijun Long, Stuart E. Denman, Christopher S. McSweeney

**Affiliations:** ^1^School of Public Health, Lanzhou University, 222 Tianshui Nanlu, Lanzhou 730000, China; ^2^School of Life Science, Lanzhou University, 222 Tianshui Nanlu, Lanzhou 730000, China; ^3^CSIRO, Agriculture Flagship, Queensland Bioscience Precinct, 306 Carmody Road, St Lucia, QLD 4067, Australia

## Abstract

Host factors are regarded as important in shaping the archaeal community in the rumen but few controlled studies have been performed to demonstrate this across host species under the same environmental conditions. A study was designed to investigate the structure of the methanogen community in the rumen of two indigenous (yak and Tibetan sheep) and two introduced domestic ruminant (cattle and crossbred sheep) species raised and fed under similar conditions on the high altitude Tibetan Plateau. The methylotrophic Methanomassiliicoccaceae was the predominant archaeal group in all animals even though* Methanobrevibacter* are usually present in greater abundance in ruminants globally. Furthermore, within the Methanomassiliicoccaceae family members from* Mmc.* group 10 and* Mmc.* group 4 were dominant in Tibetan Plateau ruminants compared to* Mmc.* group 12 found to be highest in other ruminants studied. Small ruminants presented the highest number of sequences that belonged to Methanomassiliicoccaceae compared to the larger ruminants. Although the methanogen community structure was different among the ruminant species, there were striking similarities between the animals in this environment. This indicates that factors such as the extreme environmental conditions and diet on the Tibetan Plateau might have a greater impact on rumen methanogen community compared to host differences.

## 1. Introduction

The Qinghai-Tibetan Plateau (QTP) also known as the Tibetan Plateau, covers an area of 2.5 million km^2^ and is frequently referred to as the earth's “third pole,” as it is one of the major drivers of global climatic conditions [1]. Rangelands cover more than half the total area of the plateau and sustain an enormous population of ruminants including indigenous species such as yak and Tibetan sheep [2]. The yak is considered an energy-efficient ruminant adapted to the harsh environment of the plateau and a relatively low methane producer [3–5]. Enteric fermentation and feed production are the main contributors to methane emission for ruminants and represent the largest source of greenhouse gases (GHG) from the agriculture sector [6]. In the rumen, archaea produce methane mainly from the reduction of carbon dioxide (CO_2_) and hydrogen (H_2_) that arise from bacterial fermentation [7]. Enteric methane formation makes a significant contribution to GHG emissions but also represents a loss between 2 and 12% of ingested feed energy for ruminants [8].

Studies suggest that archaeal populations in the rumen can be affected by age and species of the host, diet, season, and geographic region [7, 9, 10]. It has been reported that the yak has a rumen microbial ecosystem significantly different from that of cattle [3].

The study of factors that shape the archaeal community in the rumen could provide fundamental knowledge and lead to strategies that reduce methane emissions from these livestock. For that reason, the present study was designed to investigate the structure of the methanogen community in the rumen of two indigenous (yak and Tibetan sheep) and two introduced domestic ruminant (cattle and crossbred sheep) species raised and fed under similar extreme conditions on the QTP. To the best of our knowledge the differences in methanogen populations and mechanisms that control these changes have not been investigated in indigenous and introduced ruminants that exist under the same environmental conditions. We hypothesized that, as a result of their adaptation to the harsh QTP rangelands, indigenous yak and Tibetan sheep have coevolved with a unique rumen archaeal population that is different from introduced cattle and crossbreed sheep when examined under similar dietary conditions.

## 2. Materials and Methods

### 2.1. Animals, Diets, and Experimental Design

A total of 12 castrated male animals (3.5–4 years of age) from two indigenous and two introduced ruminant groups were used in the experiment. The ruminants used were three domesticated yak (*Bos grunniens*) (BW: 215 ± 5 kg); three domestic cattle (*Bos taurus*) (BW: 169 ± 5 kg); three Gansu Alpine Fine Wool sheep (*Ovis aries*) 65 ± 2 kg; and three Tibetan sheep (*Ovis aries*) (BW: 71 ± 3 kg). Yak and Tibetan sheep are animals indigenous to the QTP. Gansu Alpine Fine Wool sheep are a cross between Tibetan sheep and Xinjiang Fine Wool sheep while the local cattle called “Huang Niu” (Yellow Cattle) are a cross of Simmental and domesticated local cattle and have been successfully introduced to the alpine pasture area. Prior to the experimental period, all of the animals were cograzing a late summer pasture on the QTP in China which comprised* Elymus nutans* and* Kobresia humilis* grasses,* Kobresia capillifolia*,* Polygonum viviparum*,* Stipa krylovii*, and* Carex moorcroftii* as the main herb species, as well as the shrubs* Salix cupularis* and* Dasiphora fruticosa*.

During the experimental period all animals were fed ad libitum a diet of oaten hay : barley (70 : 30) in group pens (oaten hay chemical composition in g/kg of DM: CP, 79; NDF, 561; ADF, 428; ash, 80; DM, 905 g/kg fresh matter; barley chemical composition in g/kg of DM: CP, 128; ash, 22; DM, 865 g/kg fresh matter). After an adaptation period of 14 days, 90 mL of rumen fluid was collected by stomach tube, filtered through four layers of sterilized gauze, immediately transferred into sterile bottles, and stored in liquid nitrogen until processing for DNA and short chain fatty acid (SCFA) analysis. All animal management and research procedures were conducted under animal use protocols approved by Lanzhou University (Uni-2010-1).

### 2.2. Short Chain Fatty Acid Analysis

Short chain fatty acid (SCFA) analysis of the rumen fluid from different samples was conducted by gas chromatography according to the method described by Guo et al. [14]. A gas chromatograph (Model 6890N/5973N, Agilent Technologies, Wilmington, DE, USA) equipped with a Flame Ionization Detector and a fused-silica capillary column (HP-20M 60 m × 0.32 mm × 0.3 *μ*m, Hewlett-Packard, Palo Alto, CA, USA) were used in this study.

### 2.3. DNA Extraction, Pyrosequencing, and Quantification

Total genomic DNA was extracted in duplicate from 300 *μ*L aliquots of thawed rumen samples (liquor and plant particles) using the QIAamp® DNA Stool kit (QIAGEN, Germany) following the manufacturer's instructions and by adding “freeze and thaw” procedure (3 times) prior to the extraction. The yield and purity of the extracted DNA were assessed using a Nanodrop Spectrophotometer 8000 (Thermo Fisher Scientific, Waltham, MA, USA) and Quant-iT dsDNA BR kit (Invitrogen, Carlsbad, CA).

A two-step 16S rRNA gene-targeted barcoded PCR as described by de Cárcer et al. [11] was used in this study with minor modifications, for Roche 454 pyrosequencing. DNA samples were normalized to 20 ng *μ*L^−1^ of PCR reaction mix (25 *μ*L) and the primary PCR targeted the 16S rRNA gene using a set of archaeal specific primers (A340F/A1000R) [12] (Table 1). The amplification consisted of an initial denaturation step at 94°C for 2 min followed by 32 cycles of denaturation at 94°C for 10 s; annealing at 57°C for 45 s; elongation at 72°C for 45 s; and a final elongation step at 72°C for 10 min. PCR products were treated with Exonuclease I and Calf Intestinal Alkaline Phosphatase enzymes (New England Biolabs, Ipswich MA) at 37°C and 80°C for 20 min each followed by a second PCR with adaptor linker and barcode primer set [11] (Table 1). The amplification was done at 95°C for 2 min followed by 10 cycles of denaturation at 95°C for 10 s; annealing at 55°C for 30 s and 68°C for 1 min; and a final extension at 68°C for 10 min. Platinum Taq DNA Polymerase High Fidelity (Invitrogen, Carlsbad, CA) was used in the primary and secondary PCR reactions.

Final PCR products were run on 1.5% agarose gels, visualised on a Gel-Doc, and bands-quantified using the Volume tool of the Quantity One software (Bio-Rad, Hercules, CA). Equimolar amounts of PCR products were pooled and gel extracted using Qiagen DNA Gel Extraction Kit (QIAGEN, Hilden, Germany). The final product was sequenced at Macrogen (Seoul, Korea) using a 454 GS FLX Sequencer (Roche, Branford, CT).

Quantitative real-time PCR (qPCR) was used for absolute quantification of the* Methanobrevibacter* population and rumen members of the Methanomassiliicoccaceae family (also known as rumen cluster C; RCC), based on copy number of target genes. Quantitative PCR was performed using the ViiA*™* 7 Real-time PCR system in 384-well optical reaction plates (Applied Biosystems, CA, USA). The primer sets used for the real-time PCR are described in Table 1. The new primers for detecting species affiliated with the* Methanobrevibacter* genus and Methanomassiliicoccaceae family were designed and analysed by the Probe Match tool of the ARB Software [15] using 16S ribosomal RNA sequence database from Greengenes [16]. The rumen Methanomassiliicoccaceae primers designed in this study were compared with a primer set developed by Jeyanathan and coworkers [13].

Validation of the specificity against target genes for the new primer sets were performed by conventional PCR (2.5 mM MgCl_2_) with Platinum Taq under the following conditions: one cycle at 94°C for 2 min, 40 cycles of 94°C for 30 s and 60°C for 15 s, and 68°C for 1 min. The PCR products from rumen samples were analysed by TA cloning (pGEM-T Easy Kit; Promega Corporation, Madison, WI) and the clones were then confirmed by sequencing using the BigDye Terminator v3.1 kit (Applied Biosystems, Foster City, CA).

Quantitative PCR assays were set up using the Platinum SYBR Green qPCR SuperMix-UDG (Invitrogen) and performed on the ViiA*™* 7 real-time PCR system. Assay conditions were optimised for primer, template DNA and MgCl_2_ concentrations, and amplicon specificity by dissociation curve analysis as described by [17]. Total microbial rumen DNA was diluted to 1 : 10 prior to use in real-time PCR assays to reduce inhibition. Each reaction (standard curves and samples) was conducted in quadruplicate.

Standard curves for absolute quantification of Methanomassiliicoccaceae and* Methanobrevibacter* were generated by dilution series (10^8^ to 10^2^ copies *μ*L^−1^) of plasmids containing the respective 16S rRNA target genes [18]. Copy numbers per microgram of sample DNA were calculated based on the plasmid copy numbers in the standards and their concentration measured by the Quant-iT kit. Abundance analysis of Methanomassiliicoccaceae was determined using Applied Biosystems ViiA*™* 7 software and then calculated according to the standard curve (*R* ^2^ > 0.99). PCR efficiency of each amplification was within a range of 97% to 104%.

### 2.4. Sequence and Statistical Analyses

Sequence data were processed using the quantitative insights into microbial ecology software package QIIME [19]. Briefly, the sequences were filtered for an average minimum quality score of 25 across a 50 bp sliding window and trimmed for length ranging from 300 bp to 600 bp. Sequences were then assigned to the different samples using their respective barcodes. Chimeric sequences were identified against the Genomes OnLine Database (Gold, version 4.0) using UCHIME [20]. Sequences were grouped into operational taxonomic units (OTUs) at 0.99 similarity threshold using uclust [21]. Error correction of 454 sequencing reads was performed using Acacia 1.52 [22]. Taxonomic assignment of rumen methanogen OTUs was performed against the Rumen and Intestinal Methanogen-DB (RIM-DB) [23]. The OTU table was subjected to *α* and *β* diversity measures using QIIME and passed through the Phyloseq and DeSeq2 R packages for further analysis [24, 25]. Heat maps and clustering of the most abundant 100 OTUs using regularized log transformed values from DeSeq2 was generated using the aheatmap function of the NMF R package [26]. Ward's minimum variance method was used for hierarchical clustering of the computed distance matrix for samples based on the Jaccard dissimilarity indices of the OTU data in the vegan package [27]. The sequences obtained in this paper have been deposited in the European Nucleotide Archive (ENA) under the accession number PRJEB13326.

Short chain fatty acids and qPCR data were subjected to one-way ANOVA analysis of SPSS version 15.0 (SPSS Inc., Chicago, IL). Treatment effects or differences were considered significant when *P* values were <0.05.

## 3. Results

### 3.1. Ruminal Fermentation

The SCFAs produced by the experimental animals are shown in Table 2. Total SCFA concentration in yak and crossbred sheep was significantly higher (*P* < 0.05) compared to cattle and Tibetan sheep. The proportion of SCFA as acetate was significantly lower (*P* < 0.05) in yak than crossbred and Tibetan sheep (71.9%, 74.1%, and 74.6%, resp.) but not different from cattle. Conversely the propionate proportion was significantly higher (*P* < 0.05) in yak and crossbred sheep than cattle and Tibetan sheep (14.5% and 15.02% versus 12.28% and 12.97%, resp.). Consequently, the acetate : propionate ratio was higher (*P* < 0.05) in cattle and Tibetan sheep (5.97 and 5.76) compared to yak and crossbred sheep (4.98 and 4.94).

### 3.2. Archaeal Community Structure

The Shannon index analysis indicated a higher diversity among Tibetan sheep (5.76 ± 0.04) and yak (5.70 ± 0.04) (*P* < 0.05) compared to crossbred sheep (5.37 ± 0.02) and cattle (5.49 ± 0.07) (Supplementary Figure  1 in Supplementary Material available online at http://dx.doi.org/10.1155/2016/5916067).

**Figure 1:**
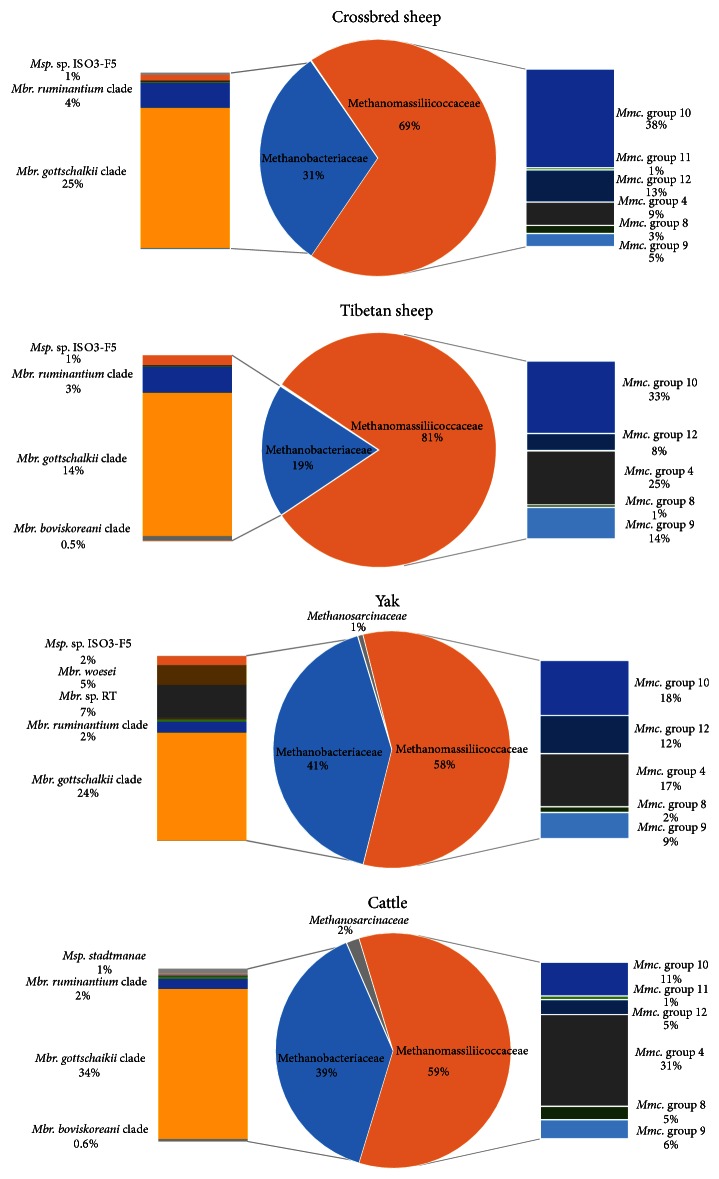
Archaeal taxonomic composition at the family level (pie chart) and species level (bar chart) for crossbred sheep, Tibetan sheep, yak, and cattle.

The taxonomic classification of the archaeal community (Figure 1) revealed the presence of the three main rumen Euryarchaeota families: the Methanobacteriaceae, Methanomassiliicoccaceae, and Methanosarcinaceae. All animals possessed a dominance of archaea populations affiliated with the Methanomassiliicoccaceae family, which was significantly higher (*P* < 0.05) in Tibetan sheep (81.1%) and crossbred sheep (69.1%) compared with yak (57.9%) and cattle (58.5%). The animals contained the five dominant Methanomassiliicoccaceae taxonomic groups, with groups 10 and 4 consistently being highly abundant across all animals (Figure 1). The second largest family belonged to the Methanobacteriaceae and this population was significantly higher (*P* < 0.05) in yak (41.3%) and cattle (39.7%) rumen compared to crossbred sheep (30.8%) and Tibetan sheep (18.7%). Within the Methanobacteriaceae family the most observed taxonomy was assigned to the* Methanobrevibacter gottschalkii *clade, followed by* Mbr. ruminantium *clade for all animals. The yak also had a high proportion of sequences affiliated with the closely related* Mbr. woesei* and* Mbr.* sp. RT clade (Figure 1). For all animals except the cattle the main* Methanosphaera* taxonomy was predominantly assigned to* Msp.* sp. ISO3-F5, while for cattle it was* Msp. stadtmanae*. The relative abundance of the Methanosarcinaceae was lower in all groups (Yak, 0.69%; cattle, 1.79%; Tibetan sheep, 0.19%; crossbred sheep, 0.01%) and was predominately assigned to taxonomy associated with* Methanimicrococcus blatticola*.

**Figure 2:**
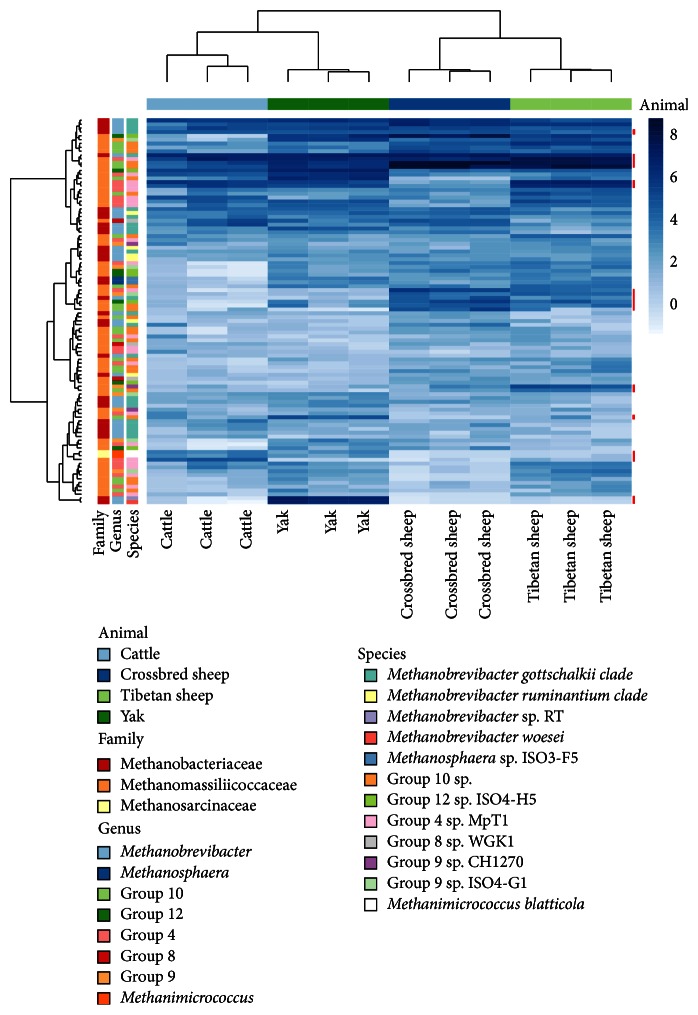
Rumen methanogen community heat maps and clustering of the most abundant 100 OTUs using regularized log transformed values from DeSeq2. Ward's minimum variance method was used for hierarchical clustering of the computed distance matrix for samples based on the Jaccard dissimilarity indices of the OTU data in the vegan package. The red bar indicates significant OTUs clustering for host species. See Supplementary Figures  3, 4, 5, 6, and 7 for further details with respect to animal pairwise comparisons.

The overall composition and relatedness of the rumen methanogen populations at the OTU level were different between animals; however, cattle and yak shared similarity and clustered together as did the crossbred sheep and Tibetan sheep (Figure 2). The beta diversity measures for the comparison of the rumen methanogen community structure showed a separation from the small ruminant and large ruminant groups explained by the first axis of variance (25.5%), while a smaller percentage of variance was observed from the cattle and yak samples along the second axis (16.1%) (Supplementary Figure  2).

The most abundant OTUs in both of the crossbred sheep and Tibetan sheep were associated with the* Mmc*. group 10, while for cattle the most abundant OTU was associated with* Mbr. gottschalkii* and for the yak the most abundant one was an OTU that was identified as being affiliated with the* Mbr. sp.* RT. In all animals the two most abundant* Mbr. gottschalkii* OTUs were the same as was their rank order. More variance in the rank order was observed for the Methanomassiliicoccaceae OTUs. However, all animals were dominated by the same* Mmc*. group 4 and* Mmc.* group 10 OTUs. Changes in the most abundant OTUs affiliated with a* Mmc.* group 12 were observed for animals with cattle and yak having different population from that of the crossbred sheep and Tibetan sheep (Figure 2). Analysis of changes in the abundance of these OTUs for specific animal pairwise comparisons is presented in the Supplementary Figures and statistically confirms the observations shown in Figure 2 (Supplementary Figures  3–8 for further details). Furthermore OTUs common to all animals as indicated in Figure 2 were not found to be significantly different in pairwise comparisons (Supplementary Figures  3–8).

The abundance of Methanomassiliicoccaceae members and* Methanobrevibacter* species as measured with qPCR is presented in Table 3 and is in agreement with the 16S sequencing data. Tibetan sheep had a significantly higher abundance (*P* < 0.05) for Methanomassiliicoccaceae archaea compared to the other ruminant groups while yak showed significantly (*P* < 0.05) more* Methanobrevibacter *spp. The two qPCR primer sets used to target* Methanomassiliicoccaceae* yielded similar estimates of abundance which were not significantly different.

## 4. Discussion

Based on the global analysis of 32 ruminant species on varying diets, the majority (~74%) of rumen archaea are members of* Mbr. gottschalkii* and* Mbr. ruminantium* clades [28]. A group of poorly understood rumen archaea affiliating with the Methanomassiliicoccaceae family together with members of the* Methanosphaera* sp. are the other dominant rumen archaea (~9%) [7, 10, 28].

While the recently published global rumen census data found the presence of Methanomassiliicoccaceae to be on average 13.5% of the archaeal population, in the present study, Methanomassiliicoccaceae was the predominant group in all animals and represented more than half of the sequences suggesting that a large population of the methanogens among the ruminants in the QTP area are yet to be functionally characterised. In addition to the higher proportion of Methanomassiliicoccaceae sequences in this study, the predominate taxonomy was also different from those reported for the global rumen census, which was mainly assigned to taxonomy from* Mmc.* group 12. For animals on the QTP the* Mmc.* group 10 and* Mmc.* group 4 dominated with significant contributions from* Mmc.* group 12 and* Mmc.* group 9. Both Tibetan and crossbred sheep presented the highest number of sequences that belong to Methanomassiliicoccaceae compared to the larger ruminants, yak and cattle, which had fewer sequences belonging to this clade. The results were in accordance with Huang et al. [3] who also found Methanomassiliicoccaceae as the dominant methanogen group in yak and cattle from the QTP. However, the abundance of Methanomassiliicoccaceae sequences in yak and cattle in that study (80.9% and 62.9%, resp.) was higher than in the current report. This could be due to factors such as the primers and sequencing method used, diet, or host factors. Other studies have also reported a high abundance of the Methanomassiliicoccaceae rumen cluster in some goats (23%) [29], cattle (63%) [30], and sheep (81%) [31].

The rumen Methanomassiliicoccaceae appears to affiliate with the methanogenic archaea which belong to the proposed new order Methanoplasmatales [32–35]. Recently, Padmanabha et al. [36] isolated a methanogen from this family and demonstrated they are obligate H_2_-dependent methylotrophic methanogens, which was also predicted from a metatranscriptomic study of rumen [34] and from genomic analysis of members of the lineage [33].

Furthermore,* Methanimicrococcus blatticola*, an obligate hydrogen dependant methylotrophic methanogen within the Methanosarcinaceae family, which utilises methanol and methylamines [37] was also observed in these animals. They were more prevalent in the cattle and yak species and nearly absent from the crossbred sheep.

The presence of methylotrophic methanogens could be indicative of diets rich in methyl compounds, for instance, with high levels of pectins or osmolytes [28]. Microbial fermentations of these plant substrates in animals on the QTP is possibly driving the delivery of higher yields of methylated compounds and thus promoting the abundance of these methylotrophic methanogens. There is a broad diversity of plants being grazed on the QTP including sedges and forbs many of which may contain these methylated compounds [2]. Further work to study changes in the bacterial populations linked to methylated compound pathways is currently being undertaken.

The second dominant archaeal group in the Tibetan ruminants belonged to the family Methanobacteriaceae (38.8–41.4 and 18.6–30.8%, for large and small ruminants, resp.), in accordance with other studies, indicating their importance in ruminants generally [3, 10, 28]. Although* Methanobrevibacter* (17–39%) and* Methanosphaera* (1-2%) were significant populations in all the Tibetan ruminants, their relative abundance was lower than reported more commonly in other ruminants (76 and 8%, resp.) [28]. Some authors have suggested a possible association between diversity of rumen methanogens and methane reduction, particularly in relation to a high abundance of Methanomassiliicoccaceae methanogens [3, 30, 38]. However, further studies are needed to confirm that lower methane emissions are characteristic of those animals that have a larger population of Methanomassiliicoccaceae related methanogens. Previously* Methanomicrobium* were reported as a major member of the rumen community with recent analyses indicating that they might represent about 5% of the methanogen population [7, 28]. Meanwhile they only presented in the crossbred sheep in a very low proportion (0.04%). Therefore, the pattern of diversity of rumen methanogens in the Tibetan ruminants was relatively similar across the four ruminant species with respect to their distinctive differences from that reported for ruminants generally. However, the populations still segregated into those from small and large ruminants which probably indicates host influences based on the size of the rumen.

Differences in abundance and presence of specific OTUs were identified and associated with specific host animals. In particular two closely related OTUs that were identified as belonging to* Mbr. woesei* and* Mbr.* sp. RT were only present in yak rumen samples and would seem to be unique to this ruminant. Both OTUs were the mainly methanogen species for the yak and not found in the other ruminants screened. Similar to this study, Huang et al. [3] also identified clones related to* Mbr. woesei* in yak and there was no report of these methanogens present in the global analysis of 32 ruminant species on varying diets [28].

The greatest diversity of archaeal OTUs was found in the indigenous populations of the QTP, the yak and Tibetan sheep. Although the cluster analysis in this study showed a different methanogen community structure in large (yak and cattle) and small ruminants (crossbred and Tibetan sheep), the dominant OTUs were shared among the four ruminant species. We conclude therefore that geographical location and dietary effects may have a greater influence on the diversity of the methanogen population than host factors. We speculate that the combination of altitude (barometric pressure), low temperatures, native plants in this extreme environment, and relative isolation of the animal populations from contact with other animals may be driving factors that sustain these microbial populations; however these hypotheses need to be tested further. This hypothesis is not in accordance with some studies which suggest a host-specific effect in methanogen community structures [13, 39]. However, apparent differences in microbial community structure can arise from the molecular techniques used to study environmental populations. At present, next generation sequencing (NGS) allows a deeper and more accurate analysis of microbial communities compared with other molecular techniques used in previous studies. This may explain why a greater number of unique OTUs were found in yak and cattle in this study compared with another report when the analysis was based on a library of about 400 clones [3].

The abundance of Methanomassiliicoccaceae was also quantified by qPCR using two primer sets, one from Jeyanathan et al. [13] and another specifically designed in the present study. Our results showed that both primer sets specifically target the Methanomassiliicoccaceae and showed a higher abundance of this family in samples from Tibetan sheep compared to cattle, yak, and crossbred sheep. The abundance of* Methanobrevibacter* in yak was significantly higher than cattle, Tibetan sheep, and crossbred sheep. These estimates of abundance using qPCR are in accordance with the relative abundance patterns provided from the 16S rRNA gene sequence analysis.

In relation to the rumen fermentation pattern, higher levels of propionate and isovaleric and total SCFA have been reported for yak [3, 40], which could represent more effective metabolic pathways for hydrogen consumption and thus decreased methanogenesis. Our results are in accordance with these studies, showing similar significant differences on the fermentation pattern in yak compared with cattle and Tibetan sheep. However, crossbred sheep exhibited a similar fermentation pattern to yak and had significantly lower acetate to propionate ratio compared with Tibetan sheep and cattle, which could be due to a crossbreeding effect, improving its adaptation to this particular environment.

In conclusion, the current study has described the rumen methanogen communities of ruminants grazing on the QTP. Although the methanogen community structure was different among the ruminant species, there were striking similarities between the animals which were distinctly different from the structure of rumen archaeal communities observed in the global rumen census [28]. This indicates that factors such as the extreme environment and diet of the QTP might have a greater impact on rumen methanogen community structure than host differences. Members of the Methanomassiliicoccaceae family were the dominant methanogen population in the ruminants from the QTP, and their specific role and function in the rumen warrant further investigation.

## Supplementary Material

The Shannon index analysis indicated a higher diversity among Tibetan sheep (5.76±0.04) and yak (5.70±0.04) (P<0.05) compared to crossbred sheep (5.37±0.02) and cattle (5.49±0.07) (Supplementary Figure 1).
The beta diversity measures for the comparison of the rumen methanogen community structure showed a separation from the small ruminant and large ruminant groups explained by the first axis of variance (25.5%), while a smaller percentage of variance was observed from the cattle and yak samples along the second axis (16.1%) (Supplementary Figure 2).
Analysis of changes in the abundance of these OTUs for specific animal pairwise comparisons is presented in the Supplementary Figures and statistically confirms the observations shown in Figure 2 (Supplementary Figures 3–8 for further details). Furthermore OTUs common to all animals as indicated in Figure 2 were not found to be significantly different in pairwise comparisons (Supplementary Figures 3–8).

## Figures and Tables

**Table 1:** Primer sets for pyrosequencing PCR and qPCR.

Name	Experiment	Target	Primer sequence (5′-3′)^a^	Reference
A304f A1000r AbcL	Pyrosequencing	Total archaea	**CGATTCATTAAAGCAGATCTCGATCCC** *CCCTAYGGGGYGCASCAG* $\underset{\text{-}\;\text{-}}{C}\underset{\text{-}\;\text{-}}{C}\underset{\text{-}\;\text{-}}{T}\underset{\text{-}\;\text{-}}{A}\underset{\text{-}\;\text{-}}{T}\underset{\text{-}\;\text{-}}{C}\underset{\text{-}\;\text{-}}{C}\underset{\text{-}\;\text{-}}{C}\underset{\text{-}\;\text{-}}{C}\underset{\text{-}\;\text{-}}{T}\underset{\text{-}\;\text{-}}{G}\underset{\text{-}\;\text{-}}{T}\underset{\text{-}\;\text{-}}{G}\underset{\text{-}\;\text{-}}{T}\underset{\text{-}\;\text{-}}{G}\underset{\text{-}\;\text{-}}{C}\underset{\text{-}\;\text{-}}{C}\underset{\text{-}\;\text{-}}{T}\underset{\text{-}\;\text{-}}{T}\underset{\text{-}\;\text{-}}{G}\underset{\text{-}\;\text{-}}{G}\underset{\text{-}\;\text{-}}{C}\underset{\text{-}\;\text{-}}{A}\underset{\text{-}\;\text{-}}{G}\underset{\text{-}\;\text{-}}{T}\underset{\text{-}\;\text{-}}{C}\underset{\text{-}\;\text{-}}{T}\underset{\text{-}\;\text{-}}{C}\underset{\text{-}\;\text{-}}{A}\underset{\text{-}\;\text{-}}{G}\underline{CAACAGCT}$ *GGCCATGCACYWCYTCTC* $\underline{\underline{CCATCTCATCCCTGCGTGTCTCCGACTCAG}}$-bc **CGATTCATTAAAGCAGATCTCGATCCC**	[11, 12]
RCC_762f RCC_1099r	qPCR	Methanomassiliicoccaceae 1	*GACGAAGCCCTGGGTC* *GAGGGTCTCGTTCGTTAT*	[13]
RCC_765f RCC_892r	qPCR	Methanomassiliicoccaceae 2	*GAAGCCCTRGGTCGCAAA* *TACTCCCCAAGTRGCMGACTT*	This study
Mbt_369f Mbt_620r	qPCR	*Methanobrevibacter*	*CCTCCGCAATGTGAGAAATCGC* *TCWCCAGCAATTCCCACAGTT*	This study

^a^Bold: linker sequence; bc: barcode; italics: group-specific sequence; underline: linking sequence; double underline: 454 adaptor A; dotted underline: 454 adaptor B.

**Table 2:** Rumen total SCFA concentration (mM) and proportions (mol 100^−1^ mol) in ruminant species.

	Yak	Cattle	Crossbreed sheep	Tibetan sheep	SE
Total SCFA	83.24^a^	58.29^b^	82.73^a^	69.75^b^	3.51
Acetate	71.95^b^	73.38^ab^	74.15^a^	74.60^a^	0.38
Propionate	14.50^a^	12.28^b^	15.02^a^	12.97^b^	0.35
Isobutyrate	1.05	1.35	0.98	1.05	0.08
Butyrate	11.16^a^	12.06^a^	9.04^b^	10.21^ab^	0.42
Isovalerate	1.28	0.79	0.81	1.17	0.09
Valerate	0.063	0.150	0.000	0.000	0.038
Acetate : propionate	4.98^b^	5.97^a^	4.94^b^	5.76^a^	0.15

^a,b^Mean values within a row with a different superscript are significantly different (*P* < 0.05).

SE: standard error.

**Table 3:** Abundance of Methanomassiliicoccaceae and *Methanobrevibacter* (log copy gene numbers g^−1^ ng DNA) in ruminant species.

Target	Yak	Cattle	Crossbreed sheep	Tibetan sheep	SE
Methanomassiliicoccaceae 1	4.31^b^	4.30^b^	4.30^b^	4.60^a^	0.04
Methanomassiliicoccaceae 2	4.44^b^	4.36^b^	4.39^b^	4.62^a^	0.04
*Methanobrevibacter *	3.62^a^	3.46^b^	3.18^c^	3.32^c^	0.05

^a–c^Mean values within a row with a different superscript are significantly different (*P* < 0.05).

SE: standard error.

## Abbreviations

QTP: Qinghai-Tibetan Plateau
qPCR: Quantitative PCR
SCFA: Short chain fatty acid
OTU: Operational taxonomic unit.
