# Supplementary material for: Methanogen Diversity in Indigenous and Introduced Ruminant Species on the Tibetan Plateau
Source: Archaea. 2016 Apr 28;2016:5916067. doi: 10.1155/2016/5916067 (PMC4864563; doi:10.1155/2016/5916067)

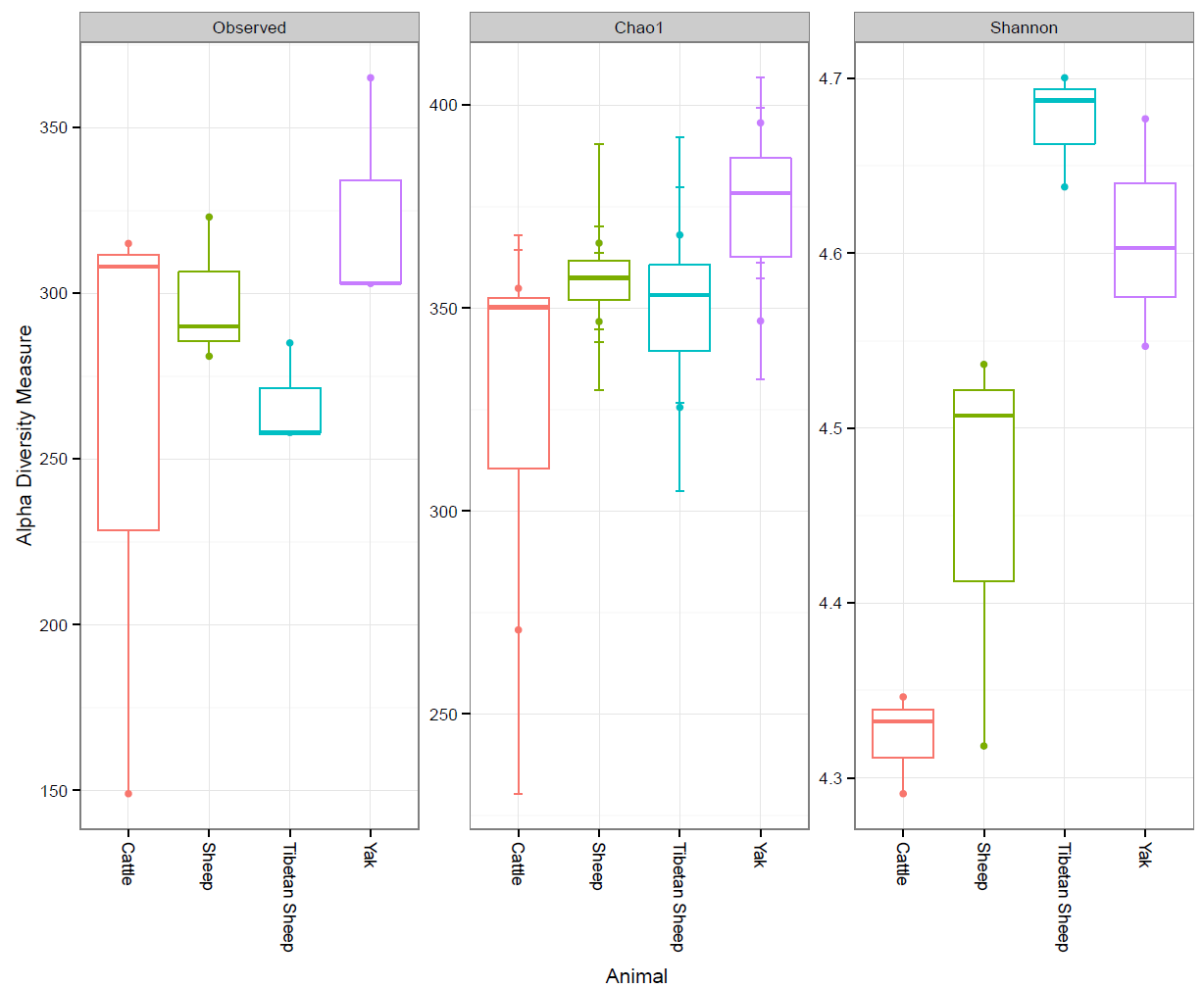
**Supplementary Figure 1.** Alpha diversity measures for host rumen methanogen communities illustrating the total observed taxonomic units (Observed), the Chao1estimates (Chao1) and the Shannon diversity index (Shannon). Boxplots indicate variance within the sampled animals with the box boundaries showing the first and third quartiles, the median value indicated as a horizontal line and the whiskers extend to 1.5 times the interquartile range.

**Supplementary Figure 2.** Weighted Unifrac diversity principal coordinate analysis of rumen methanogen communities between host species (cattle, yak, Tibetan sheep and crossbred sheep).


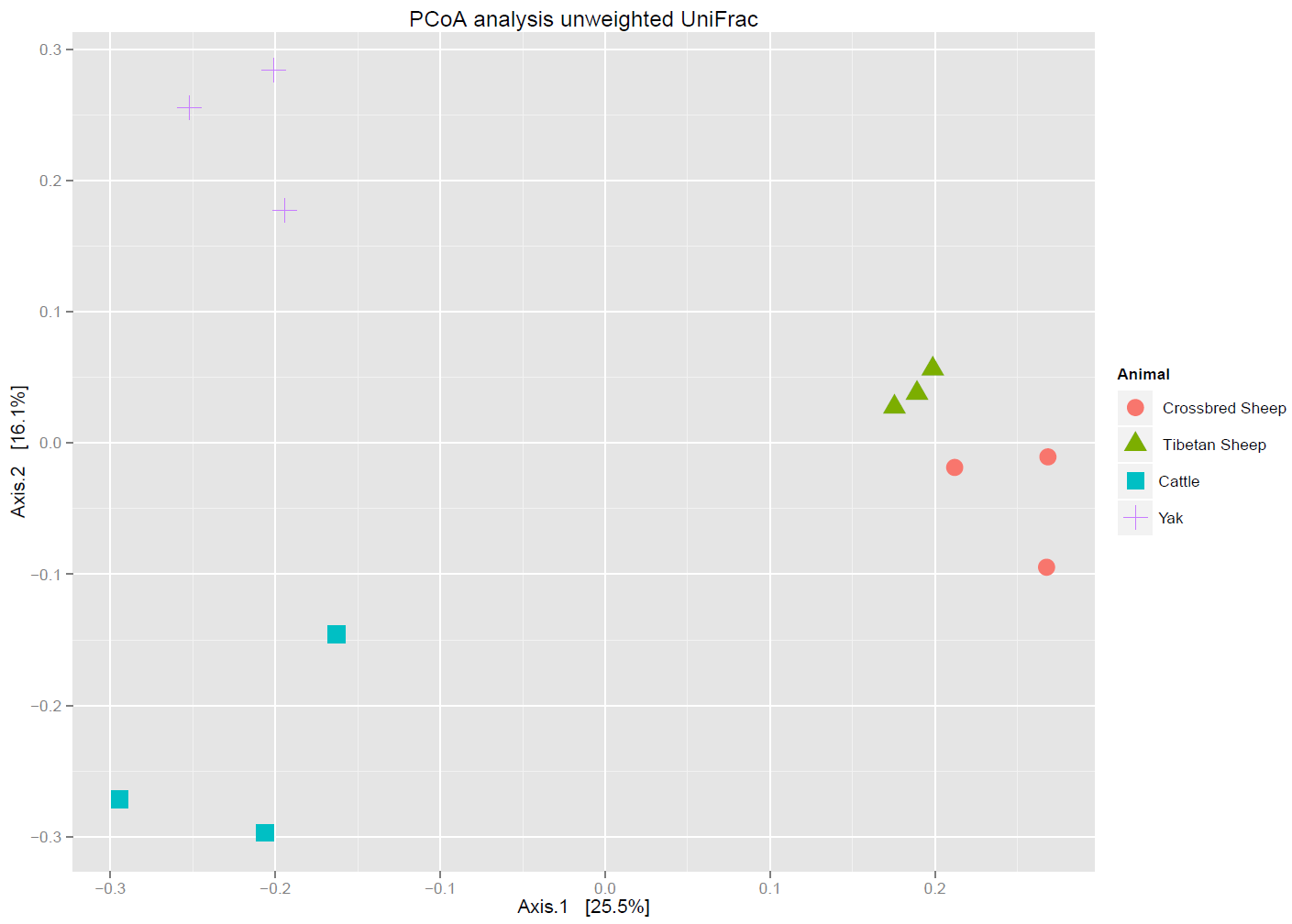


**Supplementary Figure 3.** Methanogen OTUs considered significantly different with a false discovery rate of *P* < 0.05 between yak and cattle. A positive log2-fold change value indicates that the OTU is increased that level within yak compared to cattle, and a negative log2-fold change indicates that the OTU is increased that level within cattle compared to yak. The size of the point represents the log2 transformed base mean for that OTU across the entire experiment.


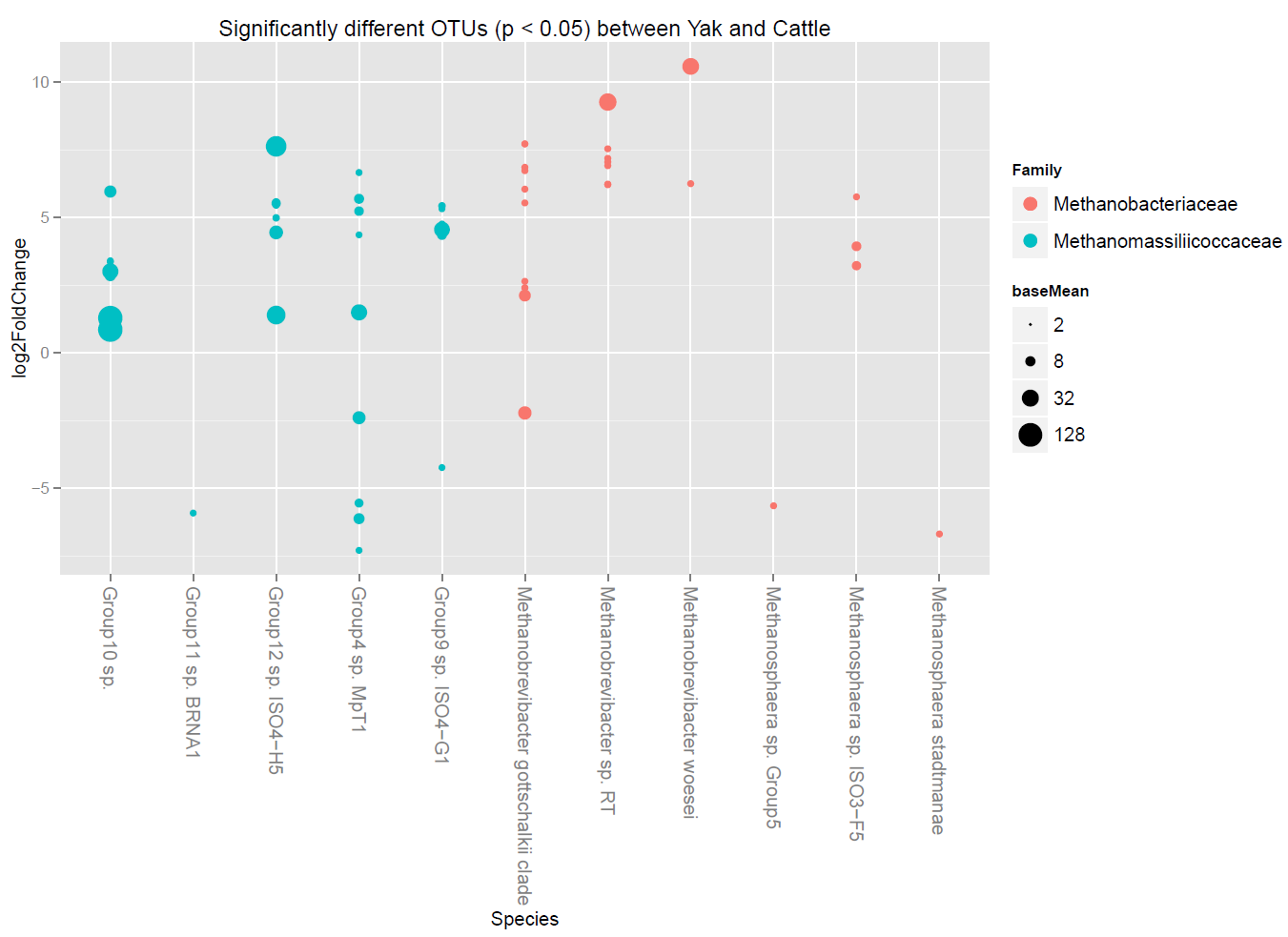


**Supplementary Figure 4.** Methanogen OTUs considered significantly different with a false discovery rate of *P* < 0.05 between yak and crossbred sheep. A positive log2-fold change value indicates that the OTU is increased that level within yak compared to crossbred sheep, and a negative log2-fold change indicates that the OTU is increased that level within crossbred sheep compared to yak. The size of the point represents the log2 transformed base mean for that OTU across the entire experiment.


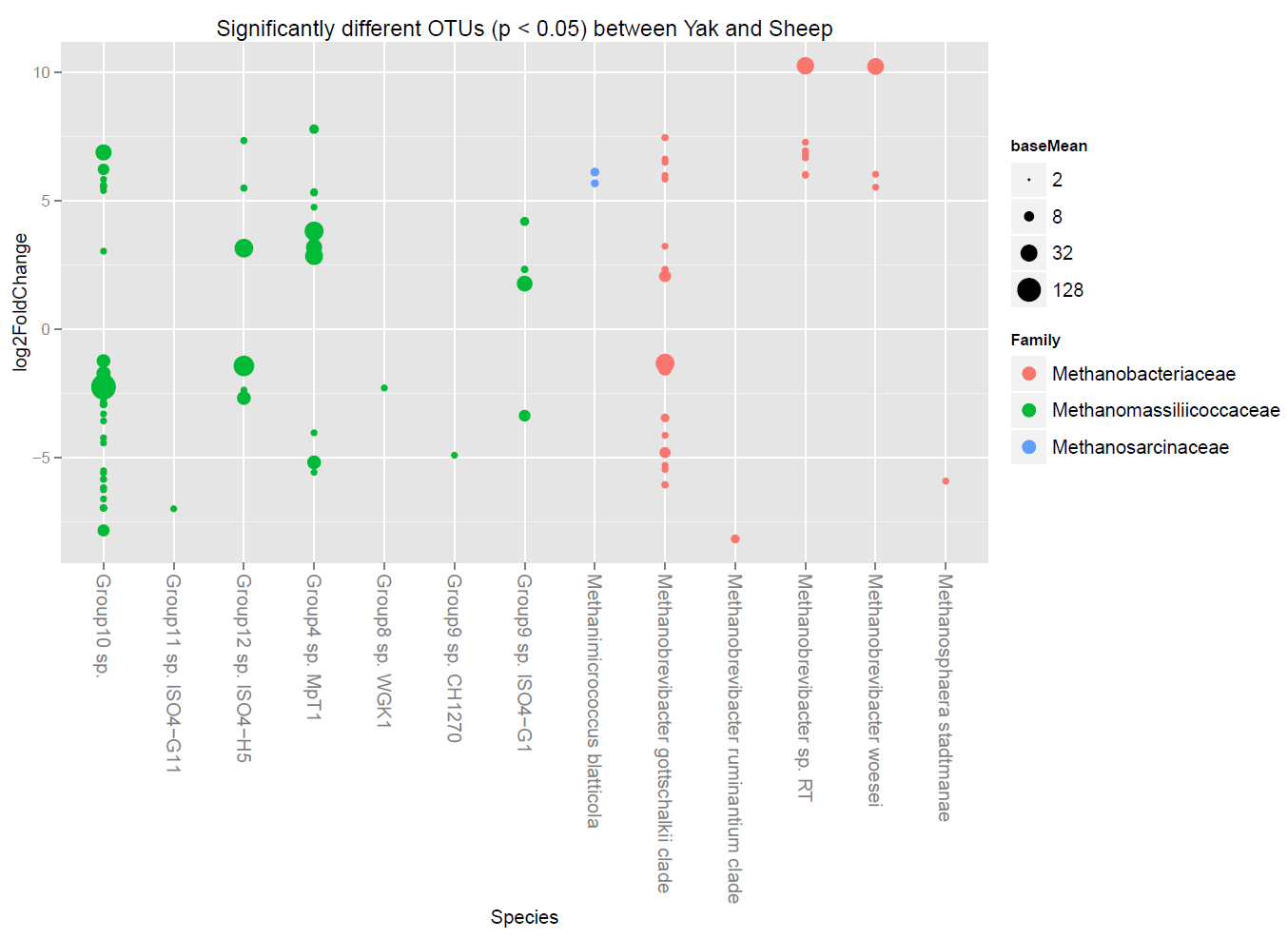


**Supplementary Figure 5.** Methanogen OTUs considered significantly different with a false discovery rate of *P* < 0.05 between yak and Tibetan sheep. A positive log2-fold change value indicates that the OTU is increased that level within yak compared to Tibetan sheep, and a negative log2-fold change indicates that the OTU is increased that level within Tibetan sheep compared to yak. The size of the point represents the log2 transformed base mean for that OTU across the entire experiment.


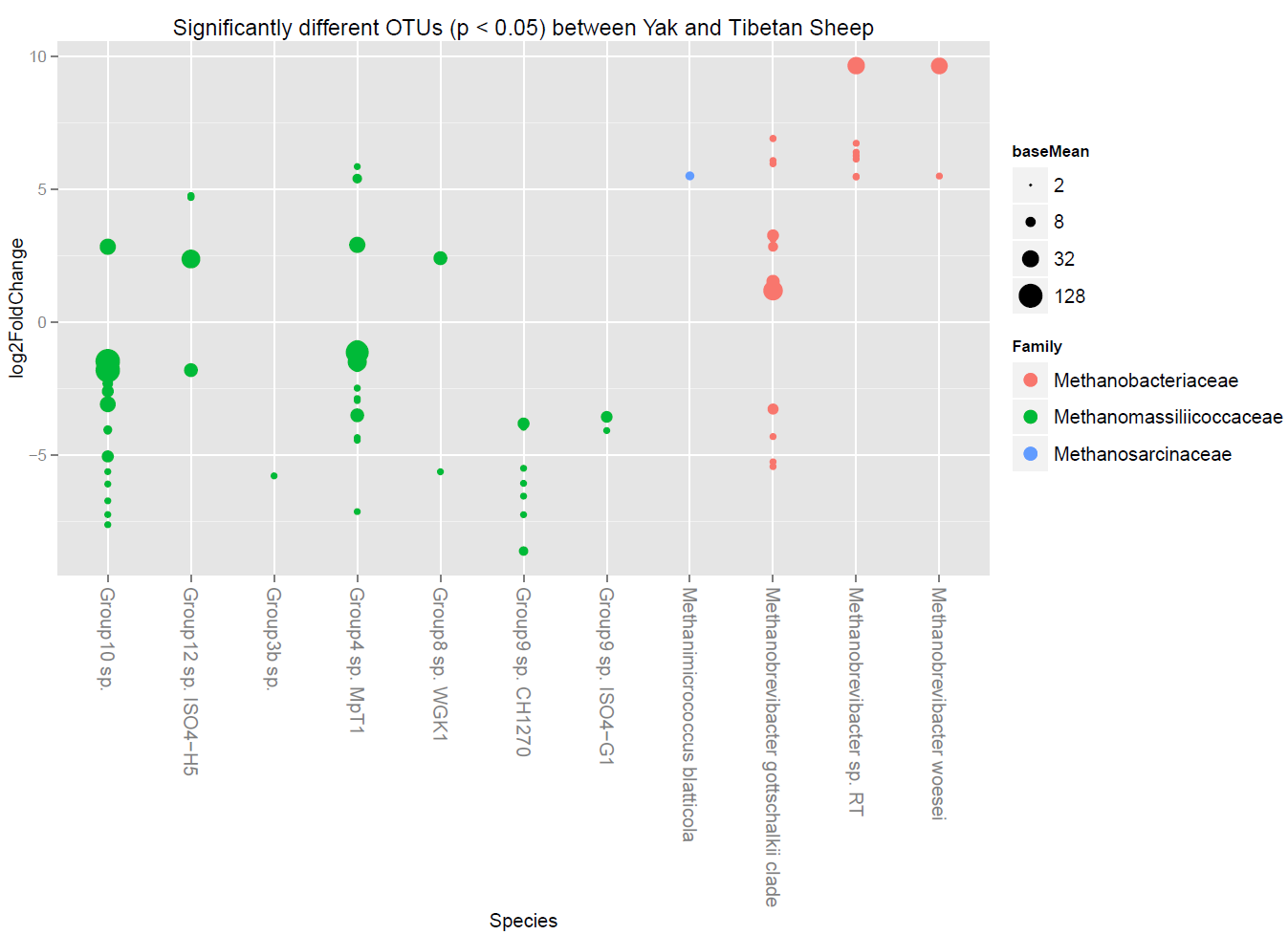


**Supplementary Figure 6.** Methanogen OTUs considered significantly different with a false discovery rate of p < 0.05 between cattle and crossbred sheep. A positive log2-fold change value indicates that the OTU is increased that level within cattle compared to crossbred sheep, and a negative log2-fold change indicates that the OTU is increased that level within crossbred sheep compared to cattle. The size of the point represents the log2 transformed base mean for that OTU across the entire experiment.


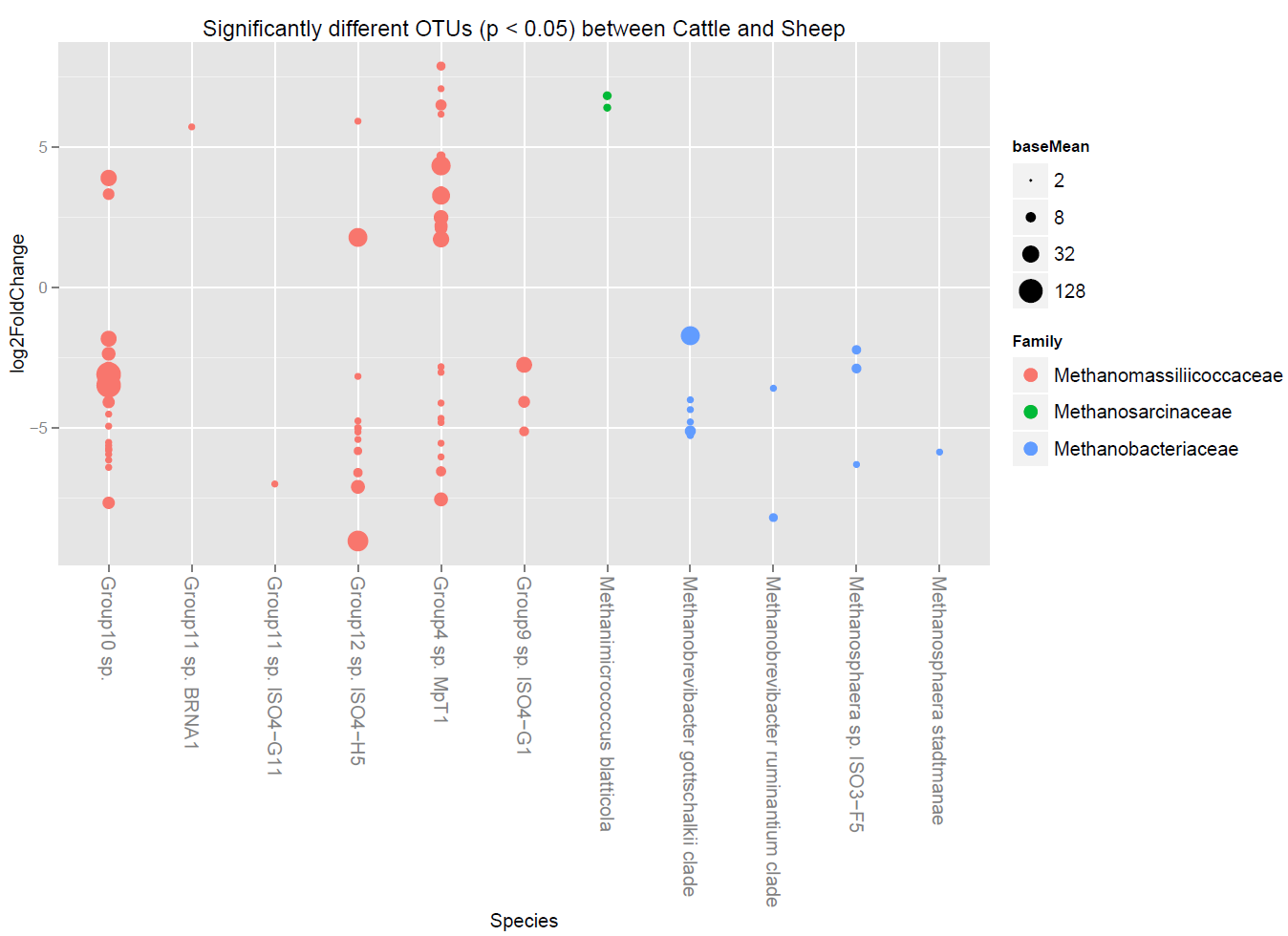


**Supplementary Figure 7.** Methanogen OTUs considered significantly different with a false discovery rate of p < 0.05 between cattle and Tibetan sheep. A positive log2-fold change value indicates that the OTU is increased that level within cattle compared to Tibetan sheep, and a negative log2-fold change indicates that the OTU is increased that level within Tibetan sheep compared to cattle. The size of the point represents the log2 transformed base mean for that OTU across the entire experiment.


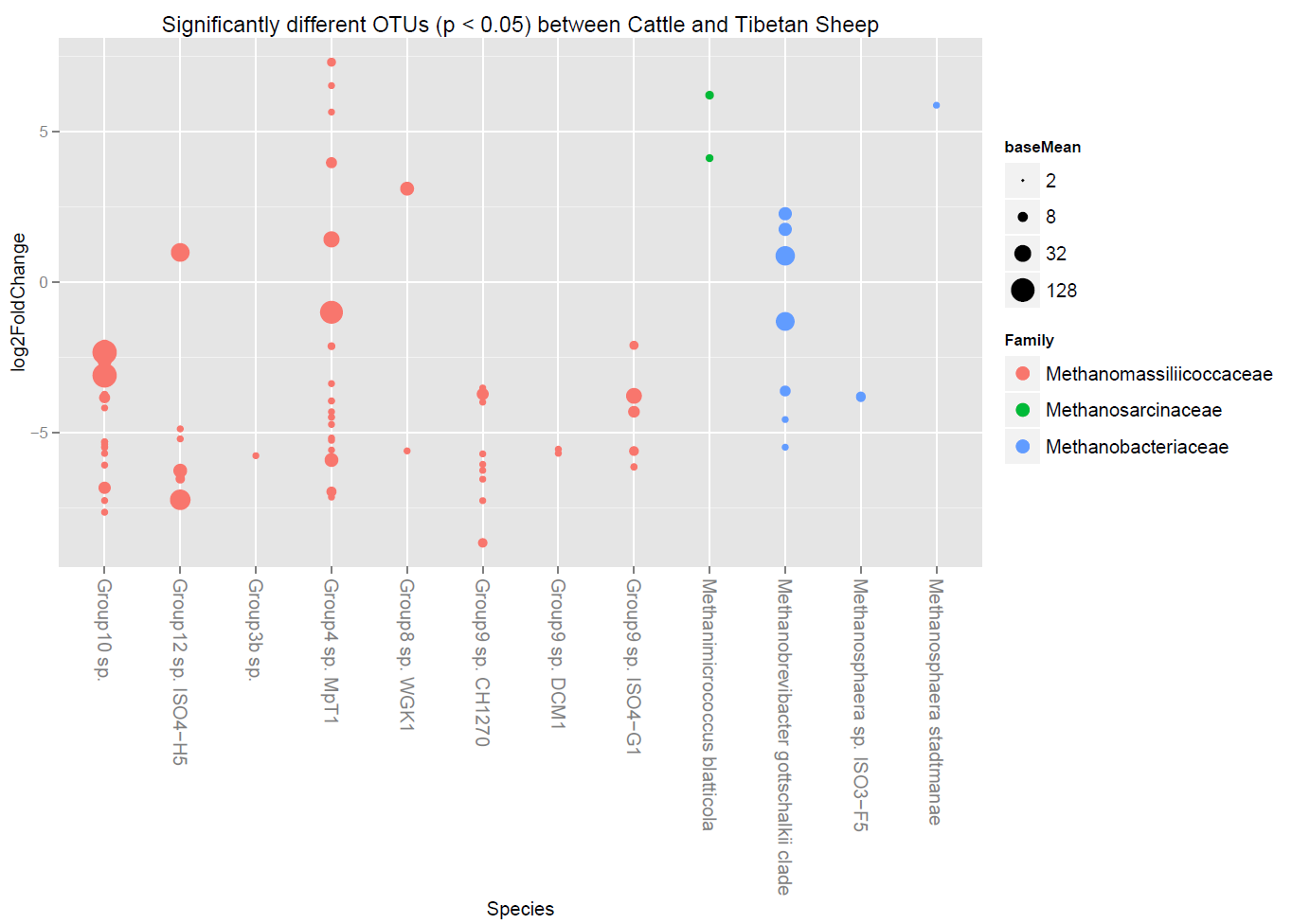


**Supplementary Figure 8.** Methanogen OTUs considered significantly different with a false discovery rate of p < 0.05 between crossbred sheep and Tibetan sheep. A positive log2-fold change value indicates that the OTU is increased that level within crossbred sheep compared to Tibetan sheep, and a negative log2-fold change indicates that the OTU is increased that level within Tibetan sheep compared to crossbred sheep. The size of the point represents the log2 transformed base mean for that OTU across the entire experiment.


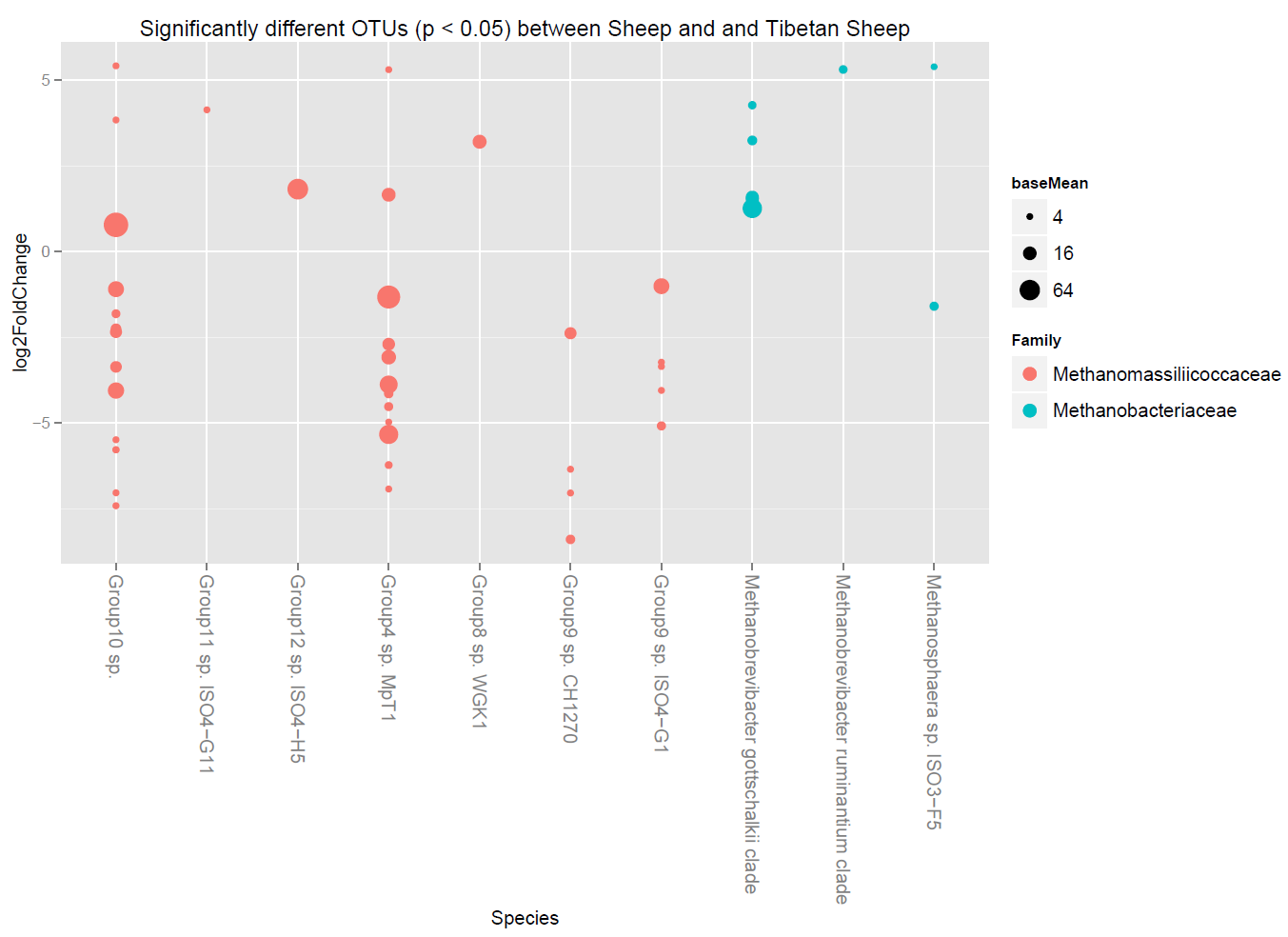

Supplement: Supplementary file 1 — The Shannon index analysis indicated a higher diversity among Tibetan sheep (5.76±0.04) and yak (5.70±0.04) (P<0.05) compared to crossbred sheep (5.37±0.02) and cattle (5.49±0.07) (Supplementary Figure 1). The beta diversity measures for the comparison of the rumen methanogen community structure showed a separation from the small ruminant and large ruminant groups explained by the first axis of variance (25.5%), while a smaller percentage of variance was observed from the cattle and yak samples along the second axis (16.1%) (Supplementary Figure 2). Analysis of changes in the abundance of these OTUs for specific animal pairwise comparisons is presented in the Supplementary Figures and statistically confirms the observations shown in Figure 2 (Supplementary Figures 3–8 for further details). Furthermore OTUs common to all animals as indicated in Figure 2 were not found to be significantly different in pairwise comparisons (Supplementary Figures 3–8). [file 5916067.f1.docx]
